# Human Endometrial CD98 Is Essential for Blastocyst Adhesion

**DOI:** 10.1371/journal.pone.0013380

**Published:** 2010-10-15

**Authors:** Francisco Domínguez, Carlos Simón, Alicia Quiñonero, Miguel Ángel Ramírez, Elena González-Muñoz, Hans Burghardt, Ana Cervero, Sebastián Martínez, Antonio Pellicer, Manuel Palacín, Francisco Sánchez-Madrid, María Yáñez-Mó

**Affiliations:** 1 Fundación IVI, Instituto Universitario IVI (IUIVI), Universidad de Valencia, Valencia, Spain; 2 Centro de Investigación Principe Felipe, Valencia, Spain; 3 Departamento de Reproducción Animal, Instituto Nacional de Investigación y Tecnología Agraria y Alimentaria, Madrid, Spain; 4 Institute for Research in Biomedicine, CIBER de Enfermedades Raras, and Department of Biochemistry and Molecular Biology, Faculty of Biology, University of Barcelona, Barcelona, Spain; 5 Servicio de Inmunología, Instituto de Investigación Sanitaria Princesa (IP), Hospital de la Princesa, Madrid, Spain; 6 Centro Nacional de Investigaciones Cardiovasculares (CNIC), Madrid, Spain; Cincinnati Children's Research Foundation, United States of America

## Abstract

**Background:**

Understanding the molecular basis of embryonic implantation is of great clinical and biological relevance. Little is currently known about the adhesion receptors that determine endometrial receptivity for embryonic implantation in humans.

**Methods and Principal Findings:**

Using two human endometrial cell lines characterized by low and high receptivity, we identified the membrane receptor CD98 as a novel molecule selectively and significantly associated with the receptive phenotype. In human endometrial samples, CD98 was the only molecule studied whose expression was restricted to the implantation window in human endometrial tissue. CD98 expression was restricted to the apical surface and included in tetraspanin-enriched microdomains of primary endometrial epithelial cells, as demonstrated by the biochemical association between CD98 and tetraspanin CD9. CD98 expression was induced in vitro by treatment of primary endometrial epithelial cells with human chorionic gonadotropin, 17-β-estradiol, LIF or EGF. Endometrial overexpression of CD98 or tetraspanin CD9 greatly enhanced mouse blastocyst adhesion, while their siRNA-mediated depletion reduced the blastocyst adhesion rate.

**Conclusions:**

These results indicate that CD98, a component of tetraspanin-enriched microdomains, appears to be an important determinant of human endometrial receptivity during the implantation window.

## Introduction

Endometrial receptivity is a self-limited period in which the endometrial epithelium acquires a functional and transient ovarian steroid-dependent status that allows blastocyst adhesion [1]. This period, termed the “implantation window”, lasts from 4–5 days to 9–10 days after progesterone production or administration. The receptive window in humans is thus limited to days 19–24 of the menstrual cycle [2].

To become receptive, the endometrium undergoes structural and biochemical modifications induced by specific gene regulation. The morphological changes include modifications of the plasma membrane [3] and cytoskeleton [4], [5]. The genomics of human endometrial receptivity has also been explored in natural cycles [6], [7], [8] but although different genes have been proposed to be essential for receptivity (see [9] for review), none has been found to have a clinical application, and evidence of function is in many cases lacking.

Embryonic implantation involves the sequential steps of apposition, attachment and invasion [10]. Structural changes in both players are dependant on a fine cross-talk between the maternal endometrium and the blastocyst that is essential for progress through each phase of implantation [10], [11]. Similar to the situation with leukocytes during extravasation, the first interaction seems to rely on carbohydrate-ligands of L-selectin expressed on the luminal epithelium at the time of apposition [12]. However, L-selectin-deficient mice do not have fertility problems [13]. Apart from L-selectin, the best characterized cell adhesion molecules on the luminal surface of the endometrium are αvβ3 integrin [14] and its ligand osteopontin, which has been repeatedly found in genome-wide studies of human receptive endometrium [6], [7], [8]. Mice deficient for CD147 (also known as basigin or EMPRINN) were reported to have implantation defects [15], and CD147 expression is restricted to the peri-implantation window in rat [16]; however, in humans, endometrial expression of this molecule appears to be less restricted [17]. HB-EGF expression is induced by the embryo on the luminal epithelium, ensuring growth-factor mediated crosstalk at the embryo-uterine interface [18].

Endometrial receptivity is probably not determined exclusively by the expression of selective adhesion molecules, and a series of cytoskeletal rearrangements is also likely to be important. Endometrial pinopodes are hormone-dependent structures that appear at the time of implantation at the apical membrane of the epithelial endometrium and represent sites of preferential blastocyst attachment [19]. Microvilli and specialized adhesive structures such as endothelial docking structures are enriched in tetraspanin-microdomains [20]. Mice deficient for tetraspanin CD9 display a severe fertility reduction due to sperm-egg fusion defects [21], [22], [23]; however, the function of CD9 in implantation remains undetermined.

Different *in vitro* and *ex vivo* models and culture systems have been used to mimic endometrial receptivity and implantation [24], [25], ranging from the use of endometrial cell lines [26], or endometrium explants [27] to artificial *ex vivo* uterus [28]. In this study we have employed two human endometrial cell lines distinguished by different adhesiveness. RL95-2 is a human epithelial cell line derived from a moderately differentiated endometrial adenocarcinoma [29], [30] that exhibits a more pronounced adhesiveness than any other human endometrial epithelial cell line for trophoblast-derived cells (JAR cells) [31] and mouse blastocysts [4]. HEC-1-A, in contrast, is only weakly adhesive, but exhibits a polarized distribution of integrins and a more epithelial morphology [4]. Screening of these cell lines identified up-regulated expression of CD98 and CD147 receptor molecules in the high-receptivity cell line RL95-2, and their potential function in endometrial receptivity was determined in vitro. Our biochemical, immunohistological and functional data demonstrated that CD98 is a key determinant of human endometrial receptivity, whose expression is tightly regulated during the implantation window.

## Results

### Adhesion receptor profiling of endometrial epithelial cell lines and primary endometrial epithelial cells

Adhesion experiments with mouse blastocysts showed a pronounced receptive phenotype for RL95-2 cells (81% blastocyst adhesion) and a non-receptive phenotype for HEC-1-A cells (46%); primary endometrial epithelial cells (EEC) cultured on extracellular matrix show an intermediate blastocyst adhesion rate of 67% [4].

**Figure 1:**
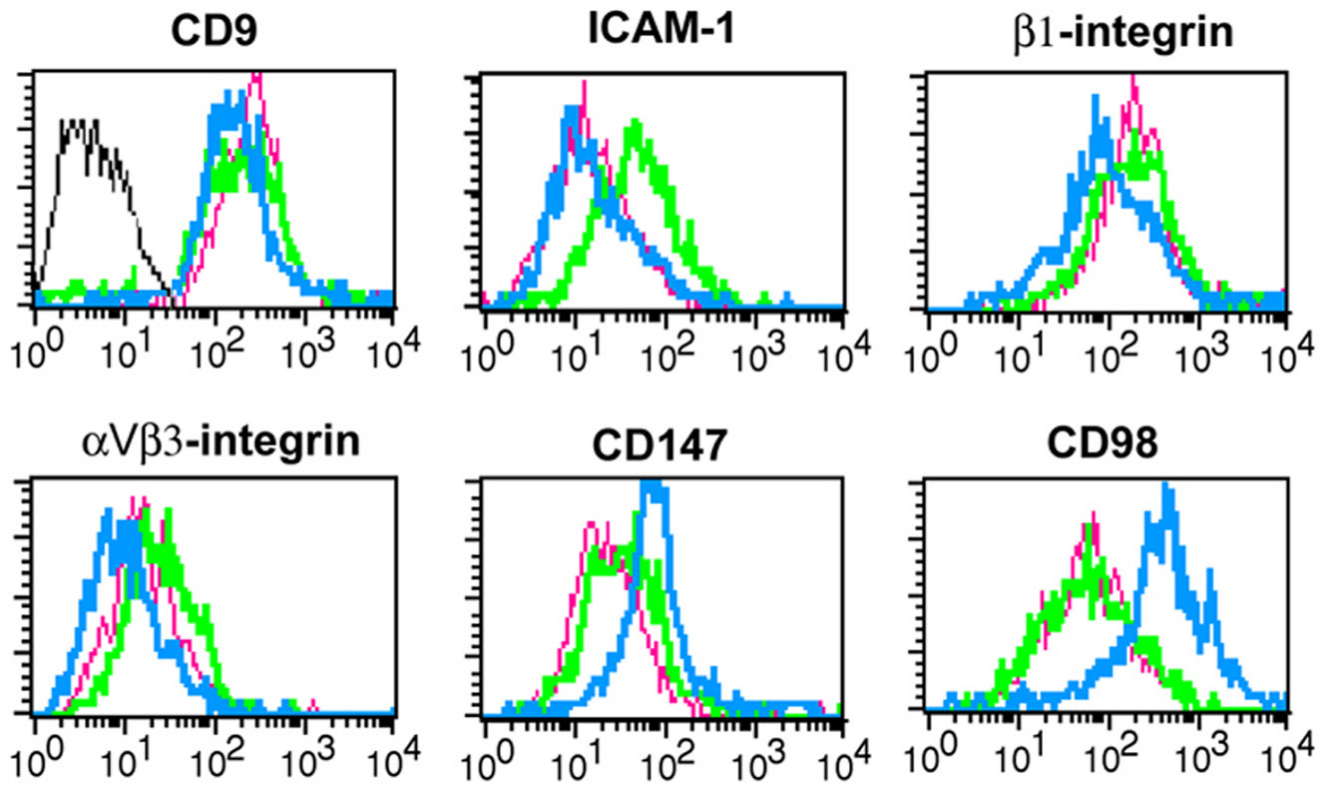
Flow cytometry analysis of the expression of cell adhesion molecules in endometrial epithelial cells and cell lines. Histogram profiles of the plasma membrane expression of CD9 (VJ1/20), ICAM-1 (HU5/3), β1 integrin (TS2/16), αvβ3 integrin (8D6), CD147 (VJ1/9) and CD98 (FG1/10) on HEC-1-A (green) and RL95-2 (blue) cell lines as well as on primary EEC cultures (pink). Negative control pX63 is shown in gray line.

In order to screen for adhesion receptors putatively involved in human endometrial adhesivity to blastocysts, we directly measured plasma membrane expression of adhesion receptors by means of a flow-cytometry based array that included specific monoclonal antibodies directed against 40 different adhesion receptors. In this analysis the fingerprint of the three epithelial endometrial cultures was overall very similar and different to that of leukocytes or endothelial cells. As shown in Table 1 and Figure 1, all three epithelial endometrial cultures expressed high levels of β1, α3 and α6 laminin-binding integrins, intermediate levels of α2, low levels of α_V_β3 integrins, and very low or undetectable levels of α1, α5, β2 or α4 integrins. The two cell lines showed a higher expression of α6 integrin compared with primary cells. In addition, all endometrial cultures expressed ICAM-1, CD44, CD59 and the tetraspanins CD9, CD81 and CD151. The cells were negative for the expression of the leukocyte markers ICAM-3, CD43 and PSGL-1, and for the endothelial molecules VCAM-1 and CD31. Endometrial cells were also negative for the expression of P, E and L selectins. In contrast, consistent with their relevance in endometrial receptivity [12], CD15, L-selectin ligands, were detected on the cell surface of both primary EEC and in a small percentage of cells from the receptive RL95-2 line, but not on HEC-1-A cells. Of all the molecules tested, the only other adhesive receptors that were more abundantly expressed in RL95-2 cells were CD98hc (heavy chain) (about 6 fold higher than in HEC-1-A and EEC cells) and CD147 (2.8 fold higher than in HEC-1-A cells and 1.4 fold higher than in EEC).

**Table 1:** Flow cytometry analysis of the expression of cell adhesion molecules in endometrial epithelial cells and cell lines.

Antigen	EEC	HEC-1-A	RL95-2
Negative control	3.2±0.9	3.6±1.9	2.2±0.8
Integrin β1	184±33	170±44	79±23* vs EEC and HEC-1-A
Integrin α1	7.8±3.2	6.3±2.4	3.7±0.37
Integrin α2	49±9	32±8	21±6
Integrin α3	46±15	52±13	22±6
Integrin α4	3.8±0.5	4.6±0.8	5±1.2
Integrin α5	6.3±1.2	11±4	2.3±0.3
Integrin α6	42±7	133±36* vs EEC	100±29
Integrin αVβ3	17±2.4	21±1.2	11±3
Integrin β2	3.2±0.4	3.8±0.2	2.2±0.5
ICAM-1	36±12	33±5	11±0.5
ICAM-2	3.3±0.2	4.3±0.5	1.8±0.3
ICAM-3	3±0.2	5.3±1.3	2±0.5
VCAM-1	3.9±0.4	5±0.75	2.7±0.7
CD31	3.8±0.3	6±2.4	2.5±0.2
CD43	3±0.4	4.8±1.5	1.8±0.7
CD44	38±15	19±8	18±3
CD59	148±10	57±10	41±5
MHC-I	394±76	45±9* vs EEC	59±4* vs EEC
E-Selectin	2.2±0.2	5.2±1.4	1.8±0.3
P-Selectin	2.7±0.3	4.3±1.1	1.3±0.1
L-Selectin	2.9±0.1	4.6±1.2	1.8±0.3
CD15	91±47	4.5±0.7	15±2.3
PSGL-1	3±0.2	5.3±1.2	2±0.2
CD147	68±27	36±8	100±12* vs EEC and HEC-1-A
CD98	50±10	54±11	307±37** vs EEC and HEC-1-A
CD9	201±50	157±44	148±32
CD81	19±2.6	109±13	20.5±0.5
CD151	32±3.8	33±8.6	10.6±0.9

Data shown are means ± s.d. of the mean fluorescence intensity (MFI) of n = 2 for integrins α4 and β1, ICAM-2 and 3; VCAM-1, CD43, Selectins, PSGL-1; n = 5 for integrins β1, α2 and α3, ICAM-1, CD147 and CD98. n = 4 for the remaining molecules. For optimal statistical analysis cytometer detectors were set to include autofluorescence basal signal into the first log of the scale. * p<0.005 **p<0.001, ANOVA Newman-Keuls Multiple Comparison Test.

### CD98 expression is restricted to the implantation window in human endometrium

**Figure 2:**
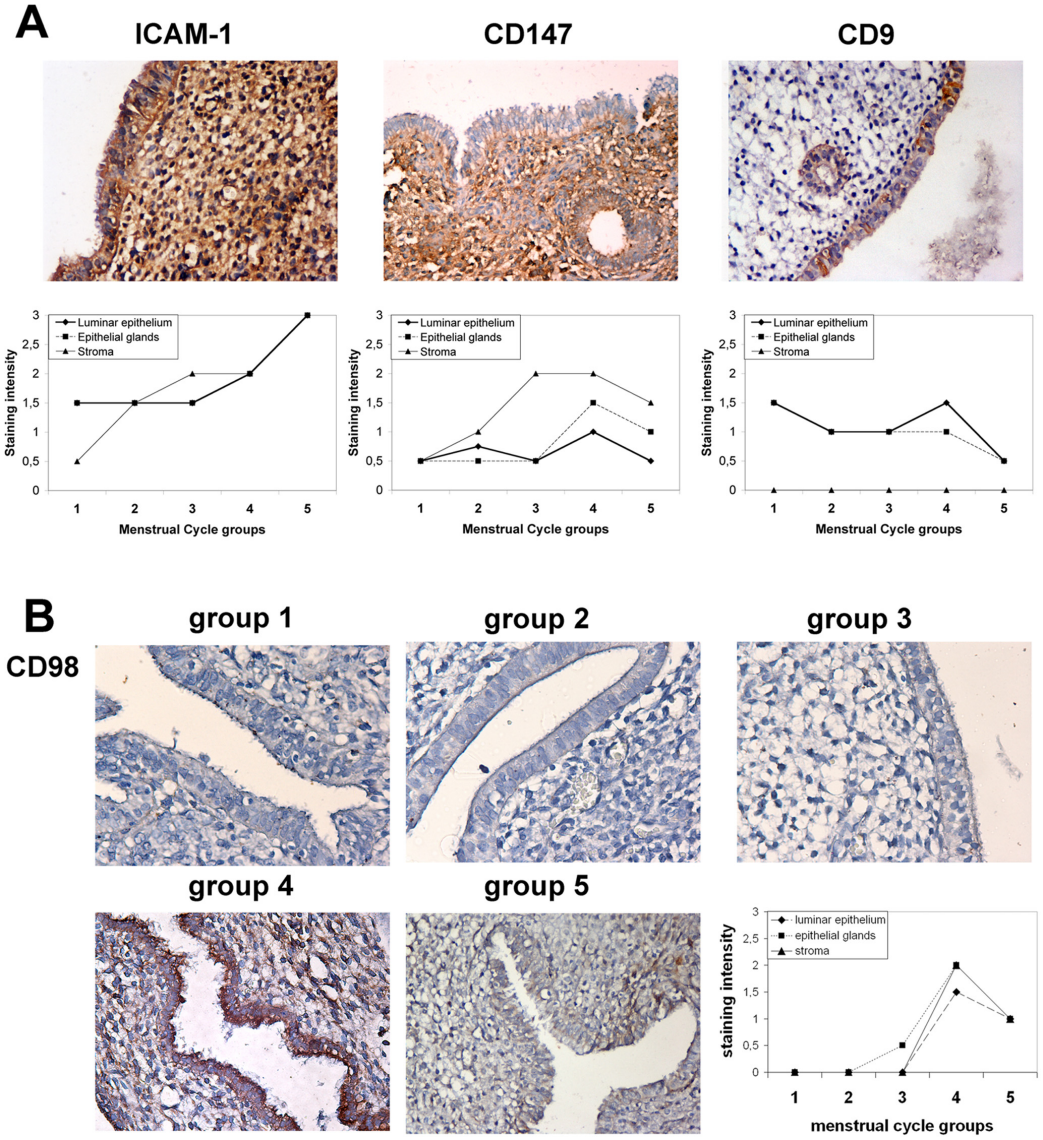
CD98 is expressed in the implantation window in human endometrium. **A**. Immunolocalization of ICAM-1 (HU5/3 mAb), CD147 (VJ1/9 mAb) and CD9 (VJ1/20 mAb) in human endometrium throughout the menstrual cycle. The micrographs show representative immunohistochemical stains of luminal epithelium in group 4 samples (day 20), corresponding to the implantation window. Images correspond to a 200× magnification. The charts plot expression based on semi-quantitative staining analysis throughout the menstrual cycle in three to five endometrial samples per group in epithelial glands (▪), luminar epithelium (♦) or stroma (▴). **B**. Immunolocalization of CD98 (anti-CD98 pAb) in human endometrium throughout the menstrual cycle. Micrographs depict the luminar epithelium in the 5 menstrual cycle groups in sequence: group1,  =  day 5, group 2 =  day9, group 3 =  day 15, group 4 =  day 20, and group 5 =  day 25. Images correspond to a 200× magnification. The chart plots semi-quantitative analysis of the stainings throughout the menstrual cycle in three to five endometrial samples per group as in A.

CD98 is a multifunctional type II glycoprotein involved in aminoacid transport [32], cell fusion [33] and integrin-dependent spreading [34], whose gene deletion is embryonically lethal [35]. CD147 deficient mice have implantation defects [15]; however, CD147 expression in humans is not so restricted to the implantation window [17]. On the other hand, tetraspanin CD9 has been also reported to associate with ICAM-1 [20] and to be expressed at the apical surface of endometrium in mice [36]. To validate the results of our in vitro model in vivo, we analyzed the expression of these adhesion molecules in human endometrium throughout the menstrual cycle. ICAM-1, CD147 and CD9 were detected in human endometrium (Figure 2A) [17], [37], [38]. ICAM-1 staining showed a high expression that peaked in the late secretory phase in both stroma and epithelium. CD147 also stained epithelial cells and its expression increased with the progression of the menstrual cycle, peaking in the mid-secretory phase (group 4) (Fig. 2A), when implantation takes place; however, a stronger staining was found on stroma. Tetraspanin CD9 staining was restricted to glandular and luminar epithelium, with no expression in the stromal compartment. No significant differences were observed throughout the menstrual cycle, with CD9 showing a weak-medium expression that declined only slightly at end of the luteal phase. Interestingly, stroma and epithelium were negative for CD98hc expression in the proliferative phase but it increased to moderately positive in the mid-secretory phase (group 4), which corresponds to the implantation window, in both luminar and glandular epithelial cells and to a lower extent in stroma, (Fig. 2B). Thus, CD9 and CD147 both occur on the luminal epithelium, while CD98 might function as a receptivity determinant, since it expression is undetectable outside the implantation window.

### CD98 is polarized to the apical surface of endometrial epithelial cells and is associated with tetraspanin-enriched microdomains

**Figure 3:**
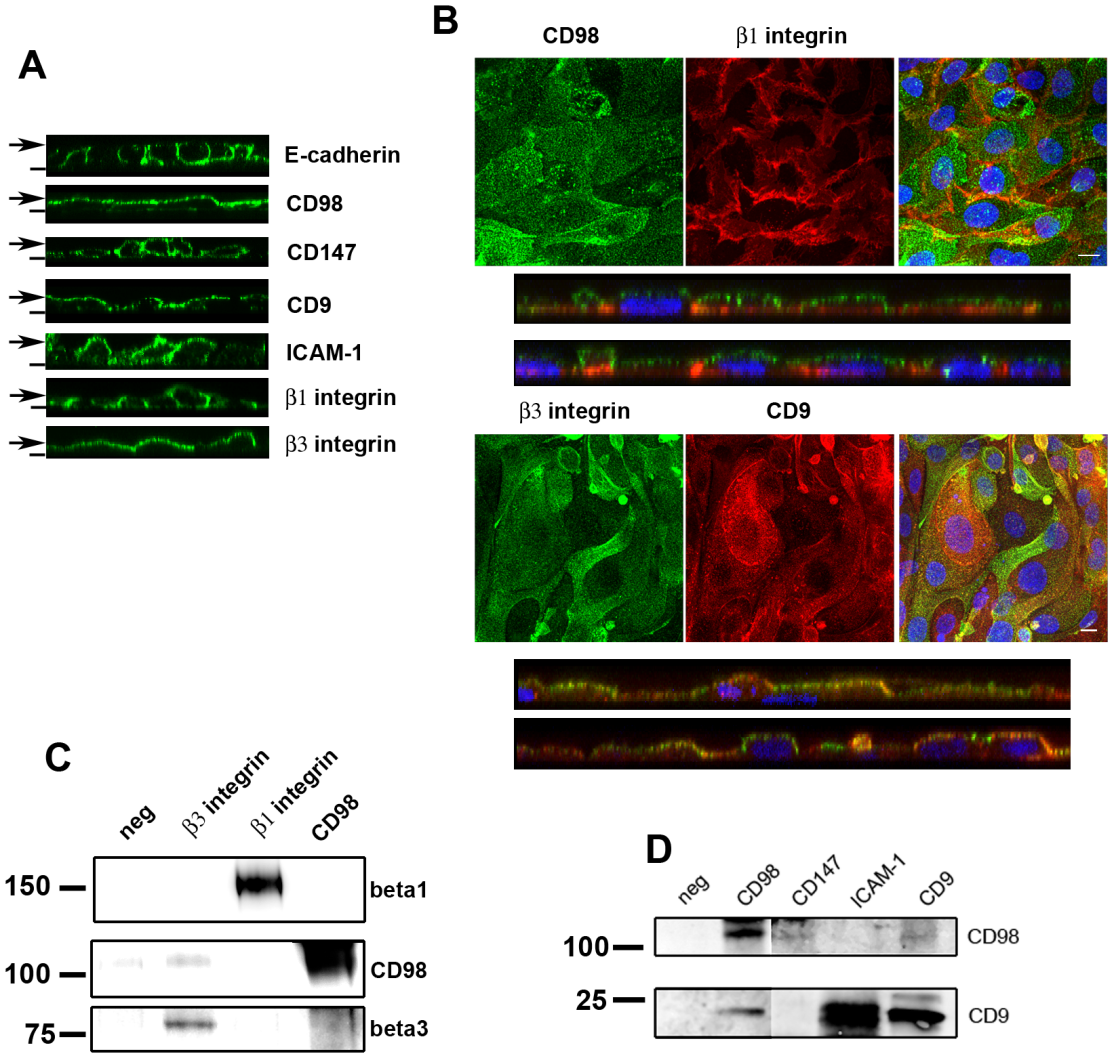
CD98 is inserted into tetraspanin-enriched microdomains on the apical surface of human endometrial cells. **A**. Confocal analysis of adhesion molecule expression in cultures of polarized human primary endometrial epithelial cells. Vertical sections were obtained with Leica Confocal Software (LASAF). Dashes mark the substratum position, whereas arrows point to the apical surface of the monolayer. **B**. Confluent EEC monolayers were double stained for CD98 (FG1/10) and β1 integrin (TS2/16) or β3 integrin (P97) with CD9 (VJ1/20) and analyzed by confocal microscopy. Nuclei were labeled with Hoechst (blue). Merged images and vertical sections of the double staining are also shown. Bars: 10 µm. **C**. Confluent EEC monolayers were lysed in buffer containing 1% Brij 96 and lysates were immunoprecipitated with anti-β3 (P97), anti-β1 (TS2/16) integrin chains mAbs or anti-CD98 serum. Non-immune Ig was included as negative control. Western blots were probed with TS2/16 for β1, and rabbit polyclonal Abs against β3 integrin and CD98. Molecular weights (kDa) are shown. **D**. Confluent EEC monolayers were lysed in buffer containing 1% Brij 96 and lysates were immunoprecipitated with anti-CD9 (VJ1/20), anti-ICAM-1 (HU5/3) or anti-CD147 (VJ1/9) mAbs or anti-CD98 serum. Non-immune Ig was included as negative control. Western blots were probed for CD9 (VJ1/20) and CD98 (anti-CD98 pAb). Different exposures of the same membrane are shown for CD98 IP. Molecular weights (kDa) are shown.

To further analyze the role of CD98 embryonic adhesion, we assessed its subcellular localization in monolayers of polarized primary EEC (confirmed by intercellular staining of E-cadherin; Fig. 3A). In contrast to what has been described in renal and intestinal epithelial cells [39], [40], CD98 is polarized to the apical surface of endometrial epithelium, colocalizing at apical microvillae with tetraspanin CD9 (Fig. 3A and B and data not shown). CD98hc apical staining was also evident in luminal epithelium in vivo (Figure 2B). ICAM-1 and CD147 were found in both the apical and the basolateral compartment. Beta1 integrins stained the basal and lateral surfaces of primary EEC, while β3 was apically expressed (Figure 3A and B).

CD98 has been shown to form molecular complexes with CD147 [41], β1 and β3 integrins [34] and ICAM-1 [39] in different cell types. We therefore analyzed the molecular association of CD98 with both β1 and β3 integrins by coimmunoprecipitation analysis in mildly restrictive conditions (1% Brij 96) (Figure 3C). Consistent with their non-overlapping subcellular localization, no association of CD98 could be detected with β1 integrin in primary EECs. Both CD98 and the β3 integrin are found in apical microvilli, and a very weak biochemical association could be detected (Figure 3C), although these data cannot be more conclusive due to the very low expression of the αVβ3 (Figure 1) or the β3 integrin chain (not shown) in our in vitro cultured cells.

Colocalization with CD9 prompted us to explore the possible insertion of CD98 in tetraspanin-enriched microdomains. Immunoprecipitation analysis in 1% Brij 96 showed a strong association of ICAM-1 with CD9 in primary endometrial epithelial cell monolayers (Fig. 3D), as previously reported for human endothelial cells [20]. In contrast, no coimmunoprecipitation of CD98 with ICAM-1 could be detected. A weaker signal could be also observed for CD9-CD98hc complexes, whereas no association with CD9 was found for CD147. The anti-CD147 mAb was able to weakly coimmunoprecipitate CD98, as previously described [41]. These results suggest that CD98 forms two independent complexes, with CD147 and with tetraspanins. Insertion into tetraspanin-enriched microdomains might regulate CD98 polarity and association with β3 integrin.

### CD98 is induced by steroidal hormones in vitro in cultured endometrial epithelial cells

**Figure 4:**
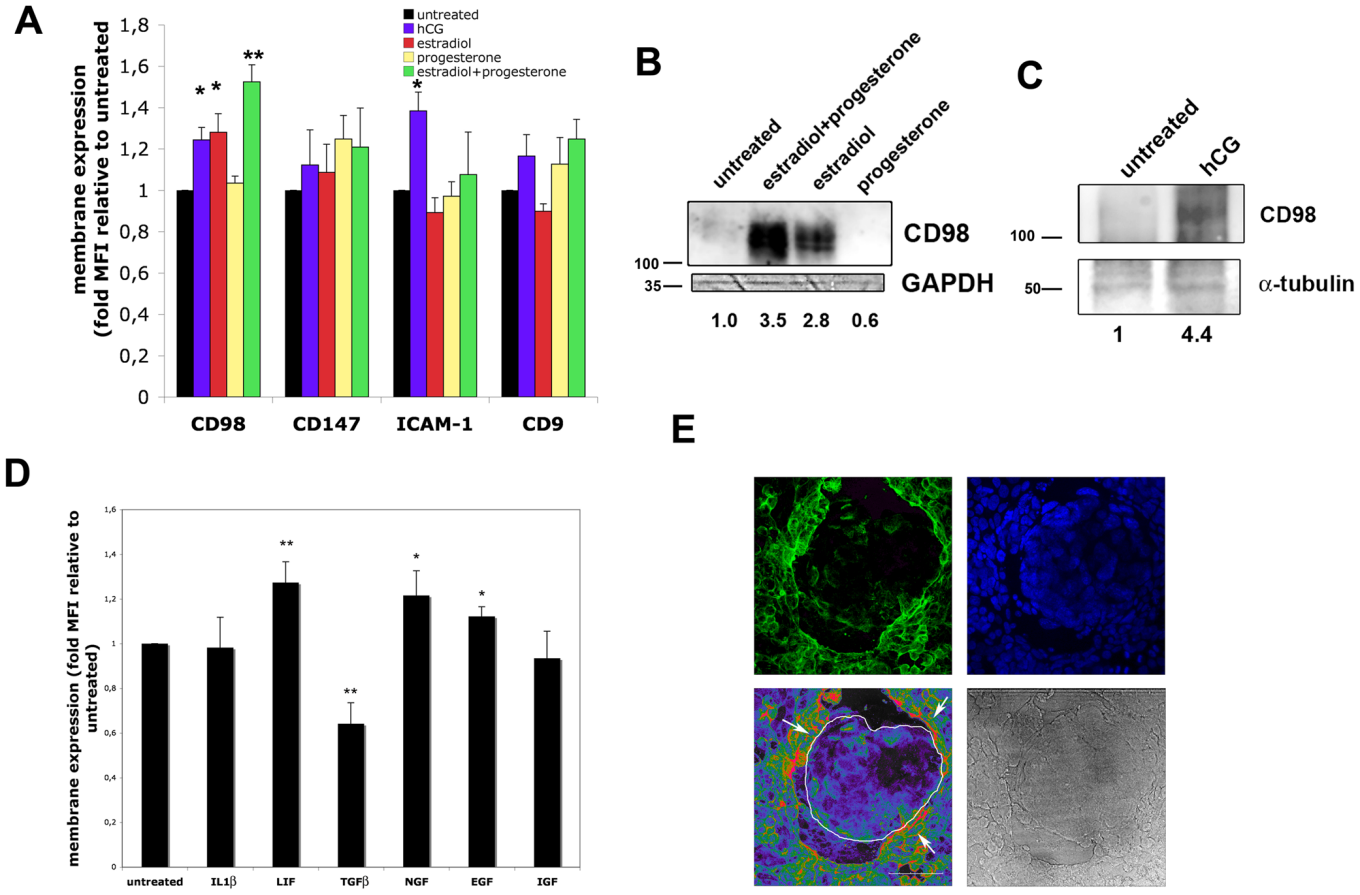
Endometrial CD98 expression is induced by hormones in vitro. **A**. Flow cytometry analysis of the membrane expression of CD98 (FG1/10 mAb), ICAM-1 (HU5/3 mAb), CD147 (VJ1/9 mAb) and CD9 (VJ1/20 mAb) in EEC cells after treatment for 48 h with hCG (100 U/ml), 17-β-estradiol (30 nM), progesterone (1 µM) or 17-β-estradiol plus progesterone. Data are the mean ± s.e.m. of the Mean Fluorescence Intensity (MFI) normalized to untreated cells in six independent primary EEC cultures. * p<0.02 ** p<0.00001 versus untreated cells in a Student T test. **B**. Western blot analysis of CD98 expression in total lysates of primary EEC after treatment for 48 h with 17-β-estradiol (30 nM), progesterone (1 µM) or 17-β-estradiol plus progesterone. GAPDH expression is shown as loading control. Numbers correspond to the densitometric analysis of CD98 protein content corrected by GAPDH loading and normalized to untreated cells. Molecular weights (kDa) are shown. **C**. Western blot of CD98 expression in total cell lysates of EEC after exposure to hCG (100 U/ml) for 48 h. α-tubulin expression is shown as loading control. Numbers correspond to the densitometric analysis of CD98 protein content corrected by tubulin loading and normalized to untreated cells. Molecular weights (kDa) are shown. **D**. Flow cytometry analysis of the membrane expression of CD98 in EEC cells after treatment for 48 h with IL1β (20 ng/ml), LIF (1000 U/ml), TGFβ (10 ng/ml), NGF (10 ng/ml), EGF (100 ng/ml) or IGF (1 µg/ml). Data are the mean ± s.e.m. of the MFI normalized to untreated cells in four independent experiments. * p<0.05 ** p<0.02 versus untreated cells in a Student T test. **E**. Mouse blastocysts were allowed to adhere to confluent HEC-1-A monolayers, and samples were fixed and stained for human CD98 (FG1/10, shown in green), and analyzed by confocal microscopy. Nuclei were labeled with Hoechst (blue). A pseudocolor image of CD98 staining intensity and a phase contrast image are shown. Mouse blastocyst is outlined on the pseudocolor image. Bar: 50 µm.

The fact that CD98hc protein is exclusively expressed on the implantation window suggests that its expression at the plasma membrane might be dictated by extracellular stimuli such as steroidal hormones. Treatment with progesterone did not significantly alter the plasma membrane expression of CD98, while exposure to 17-β-estradiol induced a significant increase of CD98hc in primary endometrial cells (Figure 4A and B). Remarkably, the greatest increment was observed upon treatment with both progesterone and 17-β-estradiol (Figure 4A and B). Human chorionic gonadotropin (hCG) treatment was also able to induce the expression of CD98 in primary EEC (Figure 4A and C). Induction of the receptive phenotype in luminal endometrial epithelium is also dictated by several paracrine factors secreted by stromal cells [11], [42]. We thus assessed CD98hc membrane expression by flow cytometry after stimulation of EEC with several soluble factors that occur *in vivo* during implantation. In these studies a marked upregulation of CD98 expression was observed after treatment of primary cells with LIF (Leukemia Inhibitory Factor), NGF (Nerve Growth Factor) and EGF (Epidermal Growth Factor). No changes were observed upon Interleukin 1β (IL-1β) treatment, while TGFβ (Transforming Growth Factor) significantly reduced CD98 membrane expression levels (Figure 4D)

We next examined the localization of CD98 in endometrial cells by confocal microscopy. Since spheroids stained strongly for CD98 expression, we performed these stainings with a blastocyst adhesion system, in which mouse blastocysts were allowed to adhere to confluent HEC-1-A cells. Samples were stained with anti-human CD98hc to only visualize endometrial CD98. CD98 expression was clearly upregulated in the endometrial epithelial cells that were in direct contact with the mouse blastocyst (Fig. 4E, arrows). Collectively, these data demonstrate the paracrine-dependent expression of CD98 in endometrial cells and the induction of the molecule by blastocyst adhesion.

### The expression level of CD98 in endometrium regulates receptivity to mouse blastocyst adhesion

**Figure 5:**
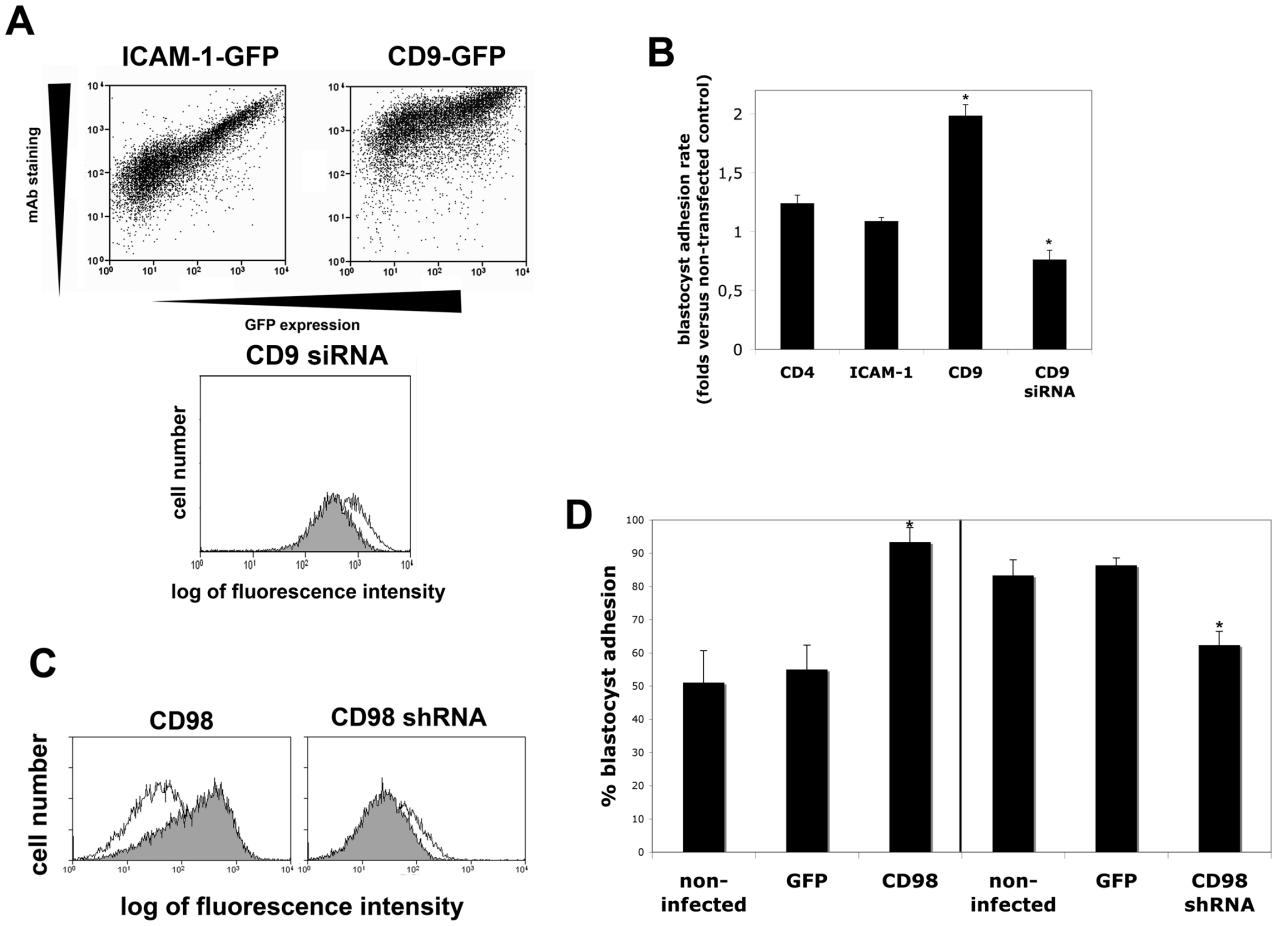
Overexpression of CD9 and CD98 enhances the receptivity of HEC-1-A cells, while their silencing impairs adhesion. **A**. HEC-1-A cells were transiently transfected with plasmid encoding the GFP-tagged versions of human ICAM-1 or CD9. 24 h after transfection cells were trypsinized and labeled with the mAb specific for the transfected receptor (HU5/3 anti-ICAM-1 or VJ1/20 anti-CD9). Dot plots of GFP versus mAb staining confirm that the GFP-expressing cells overexpress the corresponding receptor at the plasma membrane. For silencing CD9 expression, HEC-1-A cells were transfected with siRNA oligonucleotide (filled histogram), or negative control oligonucleotide (white histogram) and stained for CD9 (VJ1/20) 48 h after transfection. **B**. HEC-1-A cells were transiently transfected with GFP-tagged versions of CD4, ICAM-1 and CD9, or with CD9-targetted RNAi oligonucleotides, in independent experiments. Adhesion of mouse blastocysts to transfected cells was analyzed after co-culture for 24 or 48 h. Data are mean ± s.e.m. of three independent experiments, expressed relative to the rate of adhesion on untransfected cells in each experiment. The total number of blastocysts assessed in each condition were, CD4 n = 91, ICAM-1 n = 106, CD9 n = 169, CD9 siRNA n = 154. * p<0.05 (Mann-Whitney t test) compared with the corresponding control experiment with untransfected cells. Negative controls of adhesion ranged 50–60% for overexpression experiments analyzed at 24 h, and 70% for silencing experiments analyzed at 48 h. **C**. Flow cytometry analysis of CD98 (FG1/10) membrane expression after infection of HEC-1-A cells with lentivirus encoding human CD98 or CD98 shRNA. Expression was analyzed 72 h after infection. Expression levels in cells infected with GFP control lentivirus are shown in the white histograms. **D**. HEC-1-A cells were infected with lentivirus encoding GFP, CD98 plus GFP, or CD98 shRNA plus GFP. Three days after infection HEC-1-A monolayers were cocultured with mouse blastocysts and adhesion was quantified after 24 or 48 h. Data are mean ± s.e.m. Total number of blastocysts assessed were, non-infected n = 50, GFP n = 61, CD98 n = 56, GFP n = 39, CD98 shRNA n = 64. * p<0.05 (Mann-Whitney t test) versus GFP.

To directly assess whether CD98 was able to confer receptivity to endometrial epithelium, cells of the poorly adhesive endometrial cell line HEC-1-A were transiently transfected with GFP-tagged constructs of the different adhesion molecules. JAR spheroids or their conditioned media induced plasma membrane expression of CD98 (not shown), because of their capacity to secrete significant amounts of chorionic gonadotropin (hCG) [43]. To avoid this effect, mouse hatching blastocysts were allowed to adhere onto transfected endometrial monolayers and the number of adhered blastocysts analyzed after 24 h, or 48 h for silencing experiments to increase basal adhesion levels. Overexpression of ICAM-1 or the negative control CD4 had no significant effect on blastocyst adhesion. In contrast, overexpression of CD9 produced a significant increase in blastocyst adhesion, while siRNA mediated CD9 silencing (diminished CD9 expression down to 40% of basal expression, as determined by RT-PCR (not shown) or flow cytometry Figure 5A, note that flow cytometry data are plotted on a logarithmic scale) was able to significantly reduce blastocyst adhesion rate (Fig. 5B).

Transfection of HEC-1-A with plasmids encoding myc-tagged CD98hc, while they increased CD98 mRNA expression, did not significantly alter plasma membrane protein expression analyzed by flow cytometry. To overcome this problem, we infected cells with a lentivirus encoding human CD98hc together with a GFP infection reporter; this system achieved 2–10 fold overexpression of CD98 on the plasma membrane of HEC-1-A cells (Figure 5C). Lentiviral overexpression of CD98 produced complete adhesion of near 100% of mouse blastocysts at 24 h (Figure 5D). To directly demonstrate the role of CD98 in determining human receptivity, we reduced CD98 membrane expression by lentiviral infection with CD98-specific shRNA (30% as determined by flow cytometry, to avoid compromising cell viability, Figure 5C). Under these conditions, CD98 silencing significantly impaired mouse blastocyst adhesion (Figure 5D). All these results suggest that CD98 might be a crucial determinant of human endometrial receptivity during the implantation window.

## Discussion

Our results indicate that CD98 is an important determinant of endometrial receptivity in humans. A number of in vitro and ex vivo models and culture systems have been used to mimic the characteristics of the endometrium during the window of endometrial receptivity [24], [44] and more precisely in the process of implantation [45]. In this study we have used RL95-2 and HEC-1-A tumor endometrial-derived cell lines as appropriate models to identify markers of cellular receptivity. Since our aim was to search for adhesion molecules that could be directly involved in blastocyst adhesion, our initial screening was based on direct measurements of the levels of expression at the plasma membrane by flow cytometry. We show that the overall profile of adhesion molecule expression in these endometrial cells is comparable with that of primary endometrial epithelial cells. Some alterations, such as those affecting MHC-I, α6 integrin and CD9, might be related to the transformation process, since the expression levels are similarly altered in both cell lines when compared with primary cells. Of all the markers studied, only CD15 L-Selectin ligand [12], CD98 and CD147 were found to be increased specifically in the receptive RL95-2 cell line.

Our data show that CD98 expression in human endometrium is strictly restricted to the implantation window in a hormone dependent fashion, and its subcellular localization at the apical surface of primary endometrial epithelial cells is consistent with a role in blastocyst adhesion. Accordingly, CD98hc exogenous overexpression turned HEC-1-A into a receptive cell line. Even expanded blastocysts were found adhered to CD98 overexpressing cells, indicating that they can bind to these endometrial cells as soon as they emerge from the zona pellucida; moreover, reducing CD98 plasma membrane expression levels by 30% had a clear inhibitory effect on blastocyst adhesion. Further reduction in CD98 membrane expression could not be attained without impairing cell viability, because of the role of CD98 in aminoacid transport [40]. Analysis of the role of endometrial CD98 in implantation in mice has not been reported, since gene deletion of CD98 is embryonically lethal [35]. Mating of heterozygous animals showed empty decidual swellings, indicative of resorption after implantation. However, CD98 mRNA levels were not significantly reduced in heterozygous animals compared to wild type [35]. Posttranscriptional regulation of CD98 by miRNA has also been described [46]. Therefore, a complete deletion by conditional knockouts in endometrial epithelial tissue would help to explore this issue.

CD98 has been found associated with integrins and regulates integrin-dependent spreading [34]. CD98 expression in polarized endometrial cells is restricted to the apical surface of the endometrial cell and does not associate with β1 integrins located at the cell matrix interface. On the other hand, despite the low expression of β3 integrin in *in vitro* cultures of EEC, it may be associated to CD98 at apical microvilli. αVβ3 integrin has been shown to be specifically induced in luminal epithelium at the time of implantation [14], and therefore, proposed to play a role in this process. However, expression of this integrin did not correlate with receptivity in vitro, and it was not significantly induced in primary EEC by any of the soluble stimuli assessed (Yáñez-Mó et al., unpublished observations). Therefore, although CD98 may regulate αVβ3 function on endometrial epithelium *in vivo*, its function on endometrial adhesion to the blastocyst seems to be at least partially independent of this integrin. In other systems, CD98 has been shown to be directly involved in cell-cell interactions [47] and in this context CD98 function appears to be affected by ecto-phosphorylation [47]. Although, it is unclear in these models what the putative ligand in the counterpart cell might be, CD98 binding to galectins has been reported in other cell types [48]. Galectins, which are frequently exposed on the cell plasma membrane, bind to specific carbohydrates, which may be conserved interspecies, thus explaining our data on CD98-dependent adhesion of mouse blastocysts.

We also demonstrate here that CD98 is inserted into tetraspanin-enriched microdomains. These data are in agreement with a recent report by Wakabayashi et al.[49], that detected CD98hc in immunoprecipitates of EWI-F, an immunologbulin receptor associated to tetraspanins. In our experiments, performed in more stringent detergent conditions, the yield of CD98 in CD9 immunoprecipitates and vice versa was significantly lower than that found for ICAM-1, suggesting that the direct association of CD98 might be with a different tetraspanin protein. Mammals express 33 members of tetraspanin superfamily, but there are currently no antibodies against most of them [50]. Furthermore, CD98 has been reported to associate both with ICAM-1 [39] and with β1 integrins [51], [52]. Either or both of these molecular associations might occur in the context of tetraspanin-enriched microdomains, since these molecules also associate with tetraspanins. Association of CD98 with tetraspanins might be important for its subcellular localization in different epithelial cell types. Tetraspanin-associated ICAM-1 was suggested to be responsible for CD98 basolateral localization in intestinal epithelial cells [39]. In addition, tetraspanins are concentrated in actin-rich structures such as microvilli and are connected to ERM actin linkers [53]. Association of adhesion receptors with tetraspanins has been demonstrated to be important for avidity regulation of their adhesive functions [20], [50]. In a previous report, intrauterine injection of anti-CD9 antibodies greatly enhanced the number of implanted embryos [54]. Although the authors focused on the role of trophoblast CD9 in post-adhesion invasion steps, crosslinking of endometrial CD9 might also enhance CD98 avidity on the endometrial surface. Some tetraspanin-deficient mice have been reported to be sterile as the result of a defect in sperm-egg fusion [21], [22], [23], [55]; however, a detailed analysis of implantation was not reported. Our data reveal that overexpression of tetraspanin CD9 in HEC-1-A cells greatly enhances blastocyst adhesion. Although CD9 is constitutively expressed in luminal epithelial cells throughout the menstrual cycle, the levels of this tetraspanin in HEC-1-A were significantly lower than those of primary EEC. The restoration of CD9 levels in the transfected HEC-1-A cells might facilitate the adhesive function of associated receptors, such as CD98. Moreover, in mice CD9 expression has also been reported to be upregulated by steroid hormones and found highly expressed in epithelial cells surrounding the implanted embryo [56].

CD98 has also been proposed to be functionally linked to CD147 [41]. However, CD98 only partially colocalized with CD147 in primary endometrial epithelial cells, and appears to engage in two independent molecular interactions, with CD147 and with CD9. CD147 is strongly expressed in the mouse embryo trophoectoderm and uterine endometrium [16] and embryo transfer to the uterus of CD147 null females demonstrates that CD147 is necessary for implantation in mouse [15]. However, in humans, CD147 is expressed in both stroma and epithelium in the secretory phase [17]. We have corroborated these results, showing that CD147 is expressed in the luminar and glandular epithelium, but we also found staining in stromal cells and luminal epithelium in the proliferative phase.

In sum, our results suggest that CD98 is a putative determinant of endometrial receptivity. Moreover, the inclusion of CD98 into tetraspanin-enriched microdomains suggests potential new therapeutic approaches for the treatment of implantation failure of endometrial origin.

## Materials and Methods

### Ethics Statement

Human endometrial samples were obtained for research after written consent from patients, and the research was approved by the Institutional Review Board on the use of human subjects in research at the Instituto Valenciano de Infertilidad, and complies with Spanish Law on Assisted Reproductive Technologies (35/1988).

### Cell culture and reagents

Human endometrial carcinoma HEC-1-A cells (HTB-112), RL95-2 cells (CRL-1671) and JAR trophoblast-derived cells (HTB-144) were purchased from the American Type Culture Collection (ATCC; Rockville, MD). HEC-1-A cells were grown in McCoy 5A medium supplemented with 10% fetal bovine serum (FBS). RL95-2 cells were grown in a 1∶1 mix of Dulbecco's modified Eagles medium (DMEM) and F-12K nutrient medium, supplemented with 10% FBS.

Primary cell cultures of human endometrial epithelial cells (EEC) were derived from human endometrium samples obtained from biopsies as described [26]. Samples were obtained, after written consent (see below), in the luteal phase from fertile patients undergoing endometrial biopsy (ages 23–39 yr). Endometrial samples were minced into fragments <1 mm and subjected to mild collagenase digestion. Endometrial epithelial cells were isolated as previously described [25] and cultured to confluence in a steroid-depleted medium composed of 75% Dulbecco Modified Eagle Medium and 25% MCDB-105 (Sigma, Madrid, Spain) containing antibiotics. This medium was supplemented with 10% human albumin and 5 mg/mL insulin (Sigma). The homogeneity of the cultures was determined according to the morphologic characteristics and verified by an immunocytochemical localization of cytokeratin, vimentin, and electron microscopy scanning [25]. Confluence was reached between 4 and 6 days

17-β-estradiol, progesterone, hCG, EGF, TGFβ, NGF, IGF and IL1β were purchased from Sigma-Aldrich. LIF was from Chemicon International, Inc. (CA, USA)

### Endometrial biopsies

All samples were taken from women (ages 23–39 yr) undergoing surgery for minor gynaecological procedures using an endometrial suction curette (Pipelle, Laboratoire CCD, France). Women had no underlying endometrial pathology and had regular menstrual cycles of between 25 and 33 days. None of these women had received a hormonal preparation in the 3 months preceding biopsy collection. Endometrial samples were distributed in five groups according to the phase in the cycle: group 1, early-mid proliferative (days 1–8); group 2, late proliferate (days 9–14); group 3, early secretory (days 15–18); group 4, mid secretory (days 19–22); and group 5, late secretory (days 23–28) according to the criteria of Noyes et al [57].

### Antibodies

Monoclonal antibodies (mAbs) used were as follows: anti-β1 integrin (TS2/16), anti-α1 integrin (TS2/7), anti-α2 integrin (TEA1/41), anti-α3 integrin (VJ1/6), anti-α4 integrin (HP1/7), anti-α5 integrin (SAM-1), anti-α6 integrin (GOH3), anti-αvβ3 integrin (8D6), anti-β2 integrin (TS1/18), anti-ICAM-1 (HU5/3), anti-ICAM-2 (CBR-IC2/2), anti-ICAM-3 (HP2/19), anti-VCAM-1 (4B9), anti-CD31 (TP1/15), anti-CD43 (TP1/36), anti-CD44 (HP2/9), anti-CD59 (VJ1/12), anti-MHC-I (W6.32), anti-E-Selectin (TEA2/1), anti-P-Selectin (G1), anti-L-Selectin (Dreg55), anti-CD15 (MY1), anti-PSGL1(PL1), anti-CD147 (VJ1/9), anti-CD98hc (FG1/10), anti-CD9 (VJ1/20), anti-CD81 (I.33.2.2), and anti-CD151 (LIA1/1)[20], [58]. Anti-β3 integrin (P97) mAb was kindly donated by Dr J. González-Rodríguez (Instituto de Química Física, CSIC, Madrid, Spain).

The anti-CD98 heavy chain (CD98hc) and anti-β3 integrin rabbit polyclonal antibodies were from Santa Cruz Biotechnologies (Santa Cruz, CA) and Cell Signaling (Beverly, MA), respectively.

Negative controls used were either myeloma protein pX63 (IgG1, Kappa) or non-immune rabbit serum. Antibodies were employed at 5–10 µg/ml for all applications.

### DNA constructs and lentivirus

CD9 and ICAM-1-GFP constructs have been previously described [20]. CD4-GFP construct was from Dr M. Davis (Department of Microbiology and Immunology, Stanford University School of Medicine, CA). All HIV-1 derived lentiviral constructs (pWPXK transfer vector, pCMVΔ8,74 helper packaging construct and pMD2G vector encoding for envelope protein) were provided by Dr. D. Trono (Ecole Polytechnique Federale de Lausanne, Switzerland). The pWPXL-CD98 construct drives expression of human CD98 from the EF1alpha promoter and also contains a GFP expression cassette. CD98 shRNA was targeted to TCGGGACATAGAGAATCTGAA sequence in CD98 mRNA and inserted into pLenti6-GW-emGFP vector (Invitrogen). The lentivirus expressing EGFP is an HIV-derived third generation VSV-pseudotyped lentivirus in which EGFP expression is driven by the cytomegalovirus early promoter/enhancer. Lentivirus were produced by transient transfection into 293T cells by the calcium phosphate method, using a total of 45 µg mg of plasmid DNA on one 150 mm dish. The medium (20 ml) was replaced after 14 to 16 h. After a further 24 h, the conditioned medium was collected, cleared by low-speed centrifugation, and filtered through 0.45-mm-pore-size PVDF filters. Viral titres were calculated by measuring transduction units (TU/ml) and by qPCR of supernatants (particles/ml), and were around 10^7^ to 10^8^ TU/ml and at a ratio of 1∶100 TU/particle.

### Flow cytometry array analysis

RL95-2 and HEC-1-A human endometrial cell lines and primary EEC were trypsinized and dispensed onto 96 w plates preloaded with the different primary mAbs at a concentration of 5–10 µg/ml, followed by FITC-labelled Rabbit anti-mouse IgG (DAKO). 40 different adhesion receptors were assayed including 9 integrin chains, 5 different tetraspanins, 3 Selectins, 11 Ig receptors and 12 other adhesion molecules. Labeled cells were analyzed by flow cytometry in a FACSCanto (Becton Dickinson). Negative controls (mouse myeloma protein p3X63 (IgG1, Kappa) followed by secondary antibody and secondary antibody alone) were included as negative controls in every experiment. For optimal statistical analysis cytometer detectors were set to include autofluorescence basal signal into the first log of the scale. Trypsin treatment was routinely assessed not to affect the binding of the different antibodies employed.

When indicated, EEC confluent monolayers were treated for the indicated times with the indicated hormone stimuli before staining.

### Immunohistochemistry

Immunohistochemistry was performed on 3–5 µm endometrial sections of paraffin-embedded tissues using a DAKO LSAB Peroxidase Kit. Briefly, sections were blocked with 4% BSA for 30 min at 37°C and incubated with 3% hydrogen peroxide for 5 min at room temperature (RT) prior to incubation (60 min, RT) with primary antibodies. After 25 min incubation with the linker, streptavidin-peroxidase was added for 15 min and the substrate-cromogen solution (DAB) added for 5 min to stain the slides. Slides were counterstained with Mayer's hematoxilin. The slides were mounted in entellan (Merck, Darmstadt Germany). Immunostaining intensity was evaluated in at least three different specimens and expression scored as absent (0), weak (1), moderate (2) or strong (3) by three independent observers.

### Immunofluorescence and confocal analysis

Confluent EEC monolayers were fixed with 2% paraformaldehyde and stained with the appropriate combination of mAb and biotinylated mAb after mouse serum blocking. Confocal images were obtained with a Leica TCS-SP5 confocal scanning laser microscope and analyzed with the image processing Leica Confocal Software and Photoshop 7.0 (Adobe Systems).

### Immunoprecipitation

Confluent EEC monolayers were lysed in Tris-buffered saline containing 1% Brij 96, 1 mM CaCl_2_, 1 mM MgCl_2_ and protease inhibitors (Complete; Roche Applied Science). Cell lysates were immunoprecipitated with 5 µg of the indicated antibody coupled to protein-G-Sepharose. After washing six times with lysis buffer, proteins bound to Sepharose beads were eluted by boiling in sample buffer, separated by 10% SDS-PAGE under non-reducing conditions, and transferred onto a nitrocellulose membrane (Trans-Blot transfer medium; Bio-Rad).

### Cell transfection and infection

The cells, at 80–90% confluence, were transfected with plasmid DNA or siRNA RNA duplexes [20] using with lipofectamine 2000 (Invitrogen, Carlsbad, CA). For lentiviral infection cells were trypsinized and exposed in suspension to the lentivirus in a ratio of 100 MOI/cell before plating. Blastocyst adhesion was performed 24 h after transfection or 3 d after infection.

### Mouse blastocyst adhesion assays

Female mice of strain B6C3F1 or DBA x C57BL were used. Synchronous follicle development was triggered by intraperitoneal administration of 10 or 7.5 IU eCG (Sigma-Aldrich), followed 48 h later by 10 or 5 IU hCG (Sigma-Aldrich), respectively. Embryos were collected before natural implantation, either on day 2 post coitum and cultured for 3 days in S2 medium (Scandinavia IVF Science) or on day 3.5 and cultured for 24 h in potassium simplex optimization medium (KSOM). HEC-1-A monolayers were incubated in 2% FBS containing media 24 h before the incubation with the blastocysts and the integrity of the monolayer checked at the end of the experiment. Degenerated embryos or those with hatching defects were discarded. The zona pellucida was not artificially removed. A number of 6–10 embryos were added to confluent HEC-1A monolayers in 24 well plates and were incubated for 24 h in overexpression experiments or 48 h for silencing experiments. Attached embryos were counted under an inverted microscope [27].
